# The Mst1/2-BNIP3 axis is required for mitophagy induction and neuronal viability under mitochondrial stress

**DOI:** 10.1038/s12276-024-01198-y

**Published:** 2024-03-05

**Authors:** Dae Jin Jeong, Jee-Hyun Um, Young Yeon Kim, Dong Jin Shin, Sangwoo Im, Kang-Min Lee, Yun-Hee Lee, Dae-sik Lim, Donghoon Kim, Jeanho Yun

**Affiliations:** 1 Department of Biochemistry, College of Medicine, Dong-A University, Busan, Republic of Korea; 2 Department of Translational Biomedical Sciences, Graduate School of Dong-A University, Busan, Republic of Korea; 3 College of Pharmacy, Research Institute of Pharmaceutical Sciences, Seoul National University, Seoul, Republic of Korea; 4 Department of Biological Sciences, National Creative Research Center for Cell Plasticity, Korea Advanced Institute of Science and Technology (KAIST), Daejeon, South Korea; 5 Department of Pharmacology, College of Medicine, Dong-A University, Busan, Korea

**Keywords:** Mitophagy, Parkinson's disease

## Abstract

Mitophagy induction upon mitochondrial stress is critical for maintaining mitochondrial homeostasis and cellular function. Here, we found that Mst1/2 (Stk3/4), key regulators of the Hippo pathway, are required for the induction of mitophagy under various mitochondrial stress conditions. Knockdown of Mst1/2 or pharmacological inhibition by XMU-MP-1 treatment led to impaired mitophagy induction upon CCCP and DFP treatment. Mechanistically, Mst1/2 induces mitophagy independently of the PINK1-Parkin pathway and the canonical Hippo pathway. Moreover, our results suggest the essential involvement of BNIP3 in Mst1/2-mediated mitophagy induction upon mitochondrial stress. Notably, Mst1/2 knockdown diminishes mitophagy induction, exacerbates mitochondrial dysfunction, and reduces cellular survival upon neurotoxic stress in both SH-SY5Y cells and *Drosophila* models. Conversely, Mst1 and Mst2 expression enhances mitophagy induction and cell survival. In addition, AAV-mediated Mst1 expression reduced the loss of TH-positive neurons, ameliorated behavioral deficits, and improved mitochondrial function in an MPTP-induced Parkinson’s disease mouse model. Our findings reveal the Mst1/2-BNIP3 regulatory axis as a novel mediator of mitophagy induction under conditions of mitochondrial stress and suggest that Mst1/2 play a pivotal role in maintaining mitochondrial function and neuronal viability in response to neurotoxic treatment.

## Mst1/2: pioneering mitophagy induction amidst mitochondrial stress

Mitochondria, the energy producers of cells, are vital for cell function and energy balance. But, when stressed, they can malfunction and contribute to brain diseases like Parkinson’s and Alzheimer’s. This study found that two proteins, Mst1/2, are important for triggering mitophagy—a process that removes damaged mitochondria, in response to different mitochondrial stresses. The researchers found that Mst1/2 control this process through their kinase activity—a type of protein modification, and the stabilization of another protein, BNIP3. They also discovered that Mst1/2 are key in maintaining mitochondrial health and cell survival under conditions harmful to neurons. This research, led by Dr. Jeanho Yun and team, suggests that targeting the Mst1/2 pathway could improve mitochondrial health and protect against brain diseases.

This summary was initially drafted using artificial intelligence, then revised and fact-checked by the author.

## Introduction

Mitochondria are vital for maintaining cellular function and energy homeostasis in various cell types, including neurons^1,2^. To safeguard mitochondrial integrity and function, cells have developed intricate signaling networks to respond to diverse mitochondrial stresses, such as oxidative damage, nutrient imbalances, and mitochondrial toxins^3,4^. Failure to adequately respond to these stresses can lead to mitochondrial dysfunction, which is implicated in neurodegenerative diseases such as Parkinson’s disease and Alzheimer’s disease^5,6^. Previous studies have identified protective mechanisms against mitochondrial stress, including the regulation of mitochondrial dynamics, the unfolded protein response (UPR), antioxidant defense systems, and mitophagy^4,7^. Mitophagy, in particular, has emerged as a vital protective mechanism by promoting the removal of compromised mitochondria and recycling their components to maintain mitochondrial homeostasis^8,9^. Recent mechanistic studies have identified regulatory proteins, such as PINK1 and TBK1, that induce mitophagy upon mitochondrial stress^10,11^. However, the precise regulatory network governing mitophagy induction still requires further investigation^12,13^. Given the importance of mitophagy in preserving cellular health, discovering novel regulators that modulate mitophagy in response to diverse mitochondrial stresses is essential for understanding cellular responses to mitochondrial stresses and developing novel therapeutic strategies for neurodegenerative diseases associated with mitochondrial dysfunction.

Mst1/2, also known as Stk4 and Stk3, respectively, are serine/threonine kinases that act as central regulators in the Hippo signaling pathway, modulating the downstream transcriptional coactivators Yes-associated protein (YAP) and transcriptional coactivator with PDZ-binding motif (TAZ)^14,15^. Through the Hippo pathway, Mst1/2 kinases regulate organ size and tissue growth, prevent tumorigenesis and modulate immune responses^16^. In addition to their roles in the canonical Hippo pathway, Mst1/2 kinases have been found to have additional functions and interactions beyond the canonical Hippo pathway^17^. Previous studies have shown that Mst1/2 are activated by various cellular stresses, such as oxidative stress, DNA damage, and neurotoxic stress, and participate in the cellular response to various stressors through noncanonical processes^18–21^. Recent studies have also implicated Mst1/2 in mitochondrial functions, including mitochondrial dynamics^22,23^. However, the role of Mst1/2 in mitophagy under conditions of mitochondrial stress has not been determined.

In this study, we investigated the role of Mst1/2 in mitophagy induction upon mitochondrial stress. We found that Mst1/2 regulate the proper induction of mitophagy in response to various mitochondrial stresses through the mitophagy receptor BNIP3. While downregulated Mst1/2 expression led to exacerbated mitochondrial dysfunction, reduced cellular survival, and diminished mitophagy induction under neurotoxic treatment, Mst1 and Mst2 expression alleviated mitochondrial dysfunction and cell death in *Drosophila* and human neuronal cells. Moreover, adeno-associated virus (AAV)-mediated Mst1 expression ameliorates 1-methyl-4-phenyl-1,2,3,6-tetrahydropyridine (MPTP)-induced behavioral defects, mitochondrial dysfunction and dopaminergic neuronal death in mice.

## Methods

### Cell lines, plasmids and treatments

SH-SY5Y, HEK293 and HeLa cells were maintained in DMEM supplemented with 10% fetal bovine serum (FBS; JR Scientific, Inc., Woodland, CA, USA). HeLa cells stably expressing Flag-Parkin were generated by transfection of pcDNA3-Flag-Parkin DNA and neomycin selection. pcDNA3-Flag-Parkin and pCMV2-Flag-BNIP3 were kindly provided by Dr. Jongkyeong Chung of Seoul National University. Cell lines stably expressing mt-Keima were generated by infection with a lentivirus produced from the pLVX-mt-Keima lentiviral construct^24^. Mst1 WT (pcDNA6-Myc-His B Mst1) and Mst1 K59R (pcDNA6-Myc-His B Mst1 K59R) were kindly provided by Dr. Eui-Ju Choi of Korea University^25^. YFP-Parkin (#23955) and pCMV-Flag-Mst2 K56R (#27372) were obtained from Addgene (Watertown, MA, USA). HA-tagged Mst1 (pcDNA3-HA-Mst1) and Flag-tagged Mst2 (pcDNA3.1-Flag-Mst2) were constructed following a previously described protocol^26,27^. The construction of retrovirus expression vectors (pMSCVs) for Flag-tagged YAP WT, YAP 2SA, TAZ WT and TAZ 2SA has been previously described^28^. Carbonyl cyanide 3-chlorophenylhydrazone (CCCP), deferiprone (DFP), oligomycin, antimycin A, bafilomycin A1 (BFA) and XMU-MP-1 were purchased from Sigma‒Aldrich (St. Louis, MO, USA). 1-Methyl-4-phenylpyridinium iodide (MPP^+^) was purchased from Cayman Chemical Co. (Ann Arbor, MI, USA).

### shRNA- and siRNA-mediated knockdown

The lentiviral construct containing Mst1 shRNA has been previously described^29^. Lentiviral constructs containing Mst2 shRNA (TRCN0000002173) and control nontargeting shRNA (shNT; SHC016) were obtained from Sigma‒Aldrich. To generate additional Mst1 and Mst2 double-knockdown cells (shMst1/2 #2), we used lentiviral constructs containing Mst1 shRNA (TRCN0000001622) and Mst2 shRNA (TRCN0000002175). Mst1 and Mst2 double knockdown was also achieved by using small interfering RNAs (siRNAs) against Mst1 (L-004157-00-0010) and Mst2 (L-004874-00-0010) obtained from Dharmacon (Lafayette, CO, USA). A nonspecific siRNA (D-001206-13-20) was used as a control for siRNA transfection. For BNIP3 knockdown, lentiviral constructs containing BNIP3 shRNA (#100771) were obtained from Addgene. For PINK1 knockdown, a lentiviral construct containing PINK1 (TRCN0000199193) was obtained from Sigma‒Aldrich. The knockdown shRNA constructs were transfected into 293 L packaging cells, and the resulting cell-free viral supernatant was used to infect the cells. After puromycin selection, the resistant cells were pooled and used for the remaining experiments.

For the Mst1 rescue experiment, a nondegradable variant of Mst1 was generated by changing the Mst1 shRNA target sequence CTGAAA to TTAAAG using a QuickChange site-directed mutagenesis kit (Stratagene, La Jolla, USA) and the following primer: 5′-GCCATGGATGTGA AATTAAAGCGCCAGGAATCCCAGC-3′. The mutation was verified by sequencing.

### Measurement of mitophagy levels using an mt-Keima-based flow cytometry assay

Cellular mitophagy levels were measured using a previously established mt-Keima-based flow cytometry assay as previously described^30^. Briefly, cellular mt-Keima fluorescence was examined using an LSR Fortessa flow cytometer (BD Biosciences, Franklin Lakes, NJ, USA) equipped with 405 nm and 561 nm lasers at the Neuroscience Translational Research Solution Center (Busan, South Korea). The percentage of cells undergoing mitophagy (mitophagic cells (%)) was determined by gating cells exhibiting a high ratio of emission to excitation at 561 nm/405 nm. To distinguish between high and low emission-to-excitation ratios at 561 nm/405 nm, we used untreated HeLa-mt-Keima cells exhibiting low mitophagy activity as a standard for a low ratio. All mitophagy analyses were repeated at least three times, and the results are presented as the mean ± SD.

### Western blot analysis

Cells were lysed in RIPA buffer and subjected to western blot analysis as previously described. Anti-Mst1 (#3682), anti-Mst1 pT183 (#49332), anti-Mob1 (#3863), and anti-Mob1 pT35 (#8699) antibodies were purchased from Cell Signaling Technology (Danvers, MA, USA). Anti-Mst2 (ab52641), anti-SDHB (ab14714), anti-Cox4 (ab16056), and anti-BNIP3 (ab109362) antibodies were purchased from Abcam (Cambridge, UK). Anti-Mfn1 (SC-50330) and anti-Actin (SC-47778) antibodies were purchased from Santa Cruz (Dallas, TX, USA). An anti-PINK1 (#BC100-494) antibody was purchased from Novus Biologicals (Littleton, CO, USA). All of the western blot analyses were repeated at least three times. The band intensities were quantified using densitometry and ImageJ software (NIH).

### Electron microscopy

Ultrathin sections of cells for electron microscopic analysis were prepared at the Neuroscience Translational Research Solution Center at Dong-A University. Ultrathin sections were observed and photographed using a Talos transmission electron microscope (Thermo Fisher Scientific, Waltham, MA, USA) or an Apreo 2 S LoVac scanning electron microscope (Thermo Fisher Scientific) at the Neuroscience Translational Research Solution Center, Dong-A University. To analyze autophagosome formation upon CCCP or DFP treatment, HEK293 cells treated with CCCP or DFP were analyzed via transmission electron microscopy. At least ten cells per group were examined to determine the number of autophagosomes, and the results are presented as the mean ± SD.

### Measurement of Parkin translocation

HEK293 cells transiently expressing YFP-Parkin were generated by YFP-Parkin transfection and examined by confocal microscopy after treatment with CCCP for 2 h. To quantify Parkin translocation (% cell with Parkin translocation), the experiment was independently repeated three times, and at least sixty cells in several images per sample were analyzed in each experiment. The results are presented as the mean ± SD.

### Quantitative RT−PCR

For quantitative real-time PCR analysis, total RNA was isolated using an easy-BLUE™ Total RNA Extraction Kit (iNtRON Biotechnology, Seongnam, Korea) according to the manufacturer’s instructions, and cDNA was synthesized using TOPscript™ RT DryMIX (Enzynomics, Daejeon, Korea). Quantitative real-time PCR was performed in triplicate using SYBR Green PCR Master Mix (Enzynomics) and an ABI Prism 7500 Real-Time PCR System (Thermo Fisher Scientific). Actin was used as an internal control for all samples, and the target gene mRNA levels were normalized to the Actin RNA levels. The mRNA levels were determined using the 2^−^ΔΔCΤ threshold cycle method. For primer pairs, we used Actin-F (5′-CATGTACGTTGCTATCCAGGC-3′), Actin-R (5′-CTCCTTAATGTCACGCACGAT-3′, CT GF-F (5′-GCAGAGCCGCCTGTGCATGG-3′), CTGF-R (5‘-GGTATGTCTTCATGCTGG-3′), CYR61-F (5′-CACACCAAGGGGCTGGAATG-3′), CYR61-R (5′-CCCGTTTTGGTAG ATTCTGG-3′), ANKRD-F (5′-AGTAGAGGAACTGGTCACTGG-3′), and ANKRD-R (5′-TGT TTCTCGCTTTTCCACTGTT-3′). The experiment was independently repeated three times, and the results are presented as the mean ± SD.

### *Drosophila* strains and genotypes

*w*^*1118*^ (#60000), *control RNAi* (#60100) and *hpo RNAi* (#104169) were purchased from the Vienna *Drosophila* Resource Center (Vienna, Austria). The *appl-GAL4* (#32040), *elav-GAL4* (#458)*, elav-GS-GAL4* (#43642) and *UAS-hpo* (#44254) strains were purchased from the Bloomington Stock Center (Indiana University, Bloomington, IN). The UAS-mt-Keima line was previously generated^31^. The following genotypes were used: *appl>mt-keima, control RNAi (appl-GAL4/Y; UAS-mt-keima/+)*; *appl>mt-keima, hpo RNAi (appl-GAL4/Y; UAS-mt-keima/UAS-hpo*^*KK101704*^*); appl >mt-keima, w*^*1118*^ *(appl-GAL4/Y; UAS-mt-keima/+); appl>mt-keima, hpo (appl-GAL4/Y; UAS-mt-keima/+; UAS-hpo/+); elav>control RNAi (elav-GAL4/Y); elav>hpo RNAi (elav-GAL4/Y; UAS-hpo*^*KK101704*^*/+); elav-GS>hpo (elav-GS-GAL4/UAS-hpo); TH > mitoGFP, w*^*1118*^ *(TH-GAL4, UAS-mitoGFP/+); and TH > mitoGFP, hpo (TH-GAL4, UAS-mitoGFP/UAS-hpo)*.

### Measurement of mitophagy levels in *Drosophila*

To measure mitophagy levels in *Drosophila* brain samples, the brains of 7-day-old adult flies were dissected, and mt-Keima fluorescence was examined using a Zeiss LSM 700 confocal microscope equipped with a C-Apochromat X10/0.3 M27 lens at the Neuroscience Translational Research Solution Center (Busan, South Korea). mt-Keima fluorescence was imaged using two sequential excitation lasers (458 nm and 561 nm) and a 595–700 nm emission bandwidth. Mitophagy levels were quantified based on mt-Keima confocal images using Zeiss Zen software as previously described^24,31^. The level of mitophagy (% of mitophagy) was defined as the number of pixels with a high red/green ratio divided by the total number of pixels. At least eleven samples per group were examined to determine the level of mitophagy, and the results are presented as the mean ± SD.

### Measurement of ATP levels

To measure ATP levels in *Drosophila* brain samples, the brains of 7-day-old flies were dissected. The ten brain samples were combined, and ATP levels were measured using an ENLITEN ATP assay system kit (Promega, Mannheim, Germany) according to the manufacturer’s instructions and a LuBi microplate luminometer (Micro Digital Ltd., Seoul, Korea). The relative ATP concentration was calculated by dividing the measured ATP concentration by the total protein concentration. The protein concentration was determined by a Bradford assay (Bio-Rad, California, USA). The experiments were independently repeated four times, and the results are presented as the mean ± SD.

To measure ATP levels in the substantia nigra (SN) of mice, mitochondria were isolated from the mouse SN according to a previously established method with modifications^32,33^. Briefly, mouse brains were quickly removed and washed with ice-cold PBS, and the SN was isolated from the right hemisphere. The SN was rapidly homogenized at 4 °C, and the mitochondria were pelleted through differential centrifugation. The final mitochondrial pellet was suspended in isolation buffer to yield a final protein concentration of approximately 1 mg/ml and immediately stored on ice. The protein concentration was determined by a Bradford assay. Then, 20 μg of mitochondria was used for the ATP assay.

### *Drosophila* survival assay

To examine the effects of rotenone (Sigma‒Aldrich) on the survival of *Drosophila*, 40 male flies (7 days old) were starved for 6 h and subsequently transferred to a vial containing a rotenone medium gel (5% sucrose, 0.5% agar and 7.5 mM rotenone). Dead flies were counted at the indicated time points. The experiments were independently repeated at least three times with 40 flies per group, and the 50% survival times are shown as the mean ± SD.

### *Drosophila* dopaminergic neuron counting

To quantify dopaminergic neurons in the *Drosophila* brain, brains were dissected and imaged using a Zeiss LSM 700 confocal microscope equipped with a C-Apochromat X10/0.3 M27 lens. Dopaminergic neurons located in PPL1, PPL2, and PPM3 were counted for each brain sample. At least seven brains were analyzed for each group, and the results are shown as the mean ± SD.

### Measurement of mitochondrial membrane potential

The mitochondrial membrane potential was determined using tetramethylrhodamine methyl ester (TMRM) (Invitrogen) as previously described^34^. The TMRM fluorescence intensity was measured via flow cytometry using an LSR Fortessa cytometer. The experiments were independently repeated three times, and the results are presented as the mean ± SD.

### Cell viability assay

To assess cell viability, 1 × 10^5^ cells were seeded into 6-well plates. After treatment with MPP^+^ (500 μM) for 24 h, the numbers of viable cells and total cells were counted at the indicated time points using a LUNA automated cell counter (LogoS Biosystems, Inc., Anyang, South Korea) according to the manufacturer’s recommendation after the cells were stained with trypan blue. The experiments were independently repeated three times, and the cell viability (%) is presented as the mean ± SD.

### Mouse experiments and Mst1-specific AAV injection

C57BJ mice (7 weeks old) were obtained from SAMTAKO Bio Korea (Osan, Gyeonggi, Korea), and all procedures were performed according to a protocol approved by the Dong-A Institutional Animal Care and Use Committee (DIACUC-22-23). MPTP was purchased from Sigma‒Aldrich (St. Louis). Mice were intraperitoneally injected with MPTP (20 mg/kg, dissolved in 0.9% saline) four times a day. The control group was given an equal volume of normal saline.

A murine Mst1 expression AAV plasmid (pAAV-CMV-Flag-Mst1) was generated by subcloning Flag-Mst1 from pCMV5-Flag-Mst1 (#1965, Addgene) into the pAAV-CMV (serotype 2) vector (IBS Virus Facility, https://www.ibs.re.kr/virusfacility/). Control AAV and Mst1-AAV were produced by the IBS Virus Facility. AAV injections were administered to the SN at the following coordinates: anterior-posterior (AP) - 3.2 mm, midlateral (ML) - 1.2 mm, and dorsolateral (DV) - 4.3 mm. The values were calculated relative to the bregma according to the stereotaxic atlas below the dural surface. AAVs were injected at a volume of 2 μl at a rate of 0.2 μl every 1 min, and a total of 1 × 10^9^ genome copies (GC) were injected.

### Immunofluorescence staining and immunohistochemistry

Mst1 expression was verified four weeks after AAV injection by immunofluorescence staining. Briefly, frozen midbrain tissue was cut into 50 μm sections with a cryostat (Leica, CM3050S) and blocked in blocking buffer (10% normal goat serum, 0.02% sodium azide, 0.2% Triton X-100 in PBS) for 1 h at room temperature. Anti-Mst1 (1:100; Cell Signaling Technology, #3682) and anti-TH (1:1000; Santa Cruz, SC-25269) were used as primary antibodies, and the samples were incubated overnight at 4 °C. Sections were incubated with anti-rabbit Texas Red (Thermo Fisher Scientific) or anti-mouse FITC (Bethyl Laboratories, Montgomery, USA). The sections were visualized under an ApoTome.2 fluorescence microscope (Zeiss).

For TH immunohistochemistry and unbiased stereological counting, SN sections were blocked in blocking buffer (10% normal goat serum, 0.02% sodium azide, 0.2% Triton X-100 in PBS) for 1 h at room temperature. The sections were incubated overnight at 4 °C with rabbit polyclonal anti-tyrosine hydroxylase (TH) primary antibody in PBS containing blocking buffer (1:1000; Novus Biologicals, NB300-109). The sections were incubated with biotinylated secondary antibodies for 1 h at room temperature and incubated with ABC reagent (Vector Laboratories, Inc., Burlingame, CA, USA) for 1 h at room temperature. Immunostaining was visualized by diaminobenzidine (DAB). For Nissl double staining, TH-immunostained sections were stained with thionin before dehydration. TH^+^ cell counting and Nissl^+^ total neuron counting in the SN were conducted using Stereo Investigator software (Mbf Bioscience, Vermont, USA). A total of four sections (right hemisphere, one out of every four sections) per mouse from bregma −2.80 to −3.65 mm were collected and counted under a 40× objective. The parameters used for TH stereological counting were grid size, 200 × 200 µm; counting frame, 100 × 100 µm; and 4 µm guard zone.

### Mouse behavioral assessments

Motor coordination and balance were assessed by using a rotarod apparatus (Jeung Do Bio & Plant Co., Seoul, Korea). The mice were trained for 5 sessions per day for 3 days with a gradual increase in the rotarod speed from 8 to 30 rpm. On the rest day, the rotarod speed was set at 30 rpm, and the latency to fall was recorded up to 180 s. The mice were subjected to five trials with 30 min intervals between trials.

The pole test was implemented to evaluate motor deficits and bradykinesia using a wooden pole (10 mm in diameter and 50 cm in height). The mice were trained for 5 sessions per day for 3 days to acclimate to the pole. On the rest day, the time taken for each mouse to descend from the pole was recorded up to 60 s. The mice were subjected to five trials with 5 min intervals between trials.

## Results

### Mst1/2 are required for the proper induction of mitophagy in response to mitochondrial stress

**Fig. 1:**
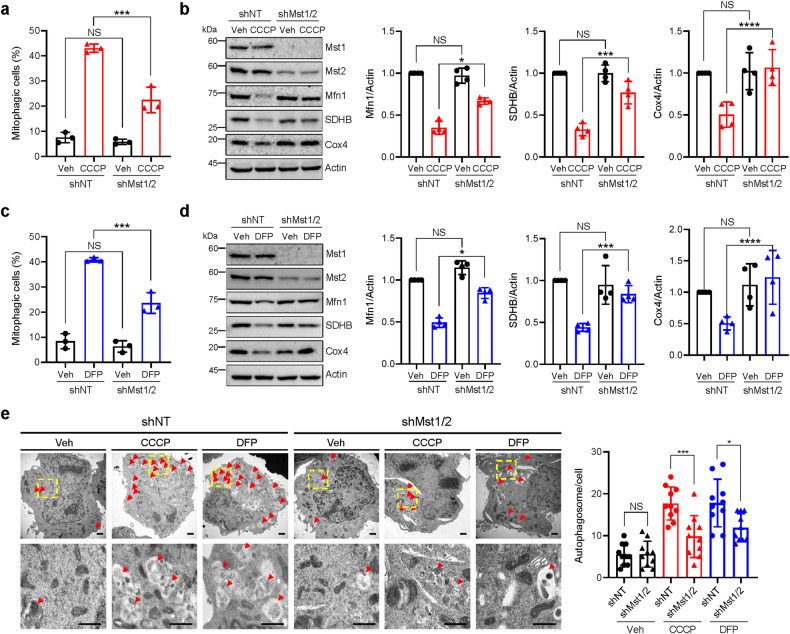
Requirement of Mst1/2 for mitophagy induction in response to mitochondrial stress. **a** HEK293 cells expressing mt-Keima and either control nontargeting shRNA (shNT) or Mst1 and Mst2 shRNAs (shMst1/2) were treated with CCCP (10 μM) for 6 h, after which mitophagy levels were analyzed via flow cytometry. **b** HEK293 cells expressing either control nontargeting shRNA (shNT) or Mst1 and Mst2 shRNAs (shMst1/2) were treated with CCCP (10 μM) for 24 h, and western blotting analysis was performed using the indicated antibodies. The results from four biological replicates are shown on the right as the mean ± SD. **c**, **d** HEK293 cells expressing mt-Keima and either control nontargeting shRNA (shNT) or Mst1 and Mst2 shRNAs (shMst1/2) were treated with DFP (1 mM) for 24 h, and mitophagy levels were analyzed via mt-Keima-based flow cytometry (**c**). Western blotting analysis was performed using the indicated antibodies (**d**). The results from four biological replicates are shown as the mean ± SD. **e** HEK293 cells expressing either control nontargeting shRNA (shNT) or Mst1 and Mst2 shRNAs (shMst1/2) were treated with CCCP (10 μM) for 6 h or DFP (1 mM) for 24 h together with bafilomycin A1 (BFA; 100 nM) and analyzed by transmission electron microscopy. Arrows indicate autophagosomes. The number of autophagosomes per cell is shown on the right as the mean ± SD (*n* = 10 per sample). Scale bars: 1 μm. Significance was determined by two-way ANOVA with Šidák’s multiple-comparison test. *P < 0.05; ***P < 0.001; ****P < 0.0001. NS not significant.

To investigate the role of Mst1/2 in mitophagy induction upon mitochondrial stress, we first generated Mst1/2 double knockdown HEK293 cells to avoid functional compensation between paralogs^35,36^ by introducing Mst1- and Mst2-specific shRNAs and verified the knockdown of Mst1 and Mst2 by western blot analysis (Supplementary Fig. 1a). Subsequently, we exposed these Mst1/2 double-knockdown cells (shMst1/2) to two different well-known mitochondrial stressors, CCCP and DFP, and then analyzed mitophagy induction using a previously established mitochondrial-targeted pH-dependent fluorescent protein Keima (mt-Keima)-based flow cytometry fluorescent-activated cell sorting (FACS) assay^24,30^. We measured mitophagy levels at earlier time points following CCCP treatment than following DFP treatment because CCCP induces mitophagy at earlier time points than DFP^37^. This experimental setup allowed us to explore the specific impact of Mst1 and Mst2 on mitophagy regulation under distinct mitochondrial challenges. CCCP disrupts the mitochondrial membrane potential, while DFP affects the respiratory chain, making them effective inducers of mitophagy^38^. Consistently, the results of the mt-Keima-based flow cytometry assay revealed that the proportion of cells actively undergoing mitophagy (mitophagic cells (%)) markedly increased in response to CCCP treatment (Fig. 1a). Interestingly, the increase in mitophagic cells in response to CCCP was abrogated by approximately 48% in shMst1/2 cells compared to control cells expressing nontargeting shRNA (shNT) (Fig. 1a). Western blot analysis also showed that the decrease in the levels of mitochondrial proteins, including Mfn1, SDHB, and Cox4, following CCCP treatment was significantly mitigated in shMst1/2 cells (Fig. 1b), suggesting that CCCP-induced mitophagy is inhibited by Mst1/2 knockdown. Similarly, we observed that the increase in the percentage of mitophagic cells and decrease in the levels of mitochondrial proteins in response to DFP treatment were also significantly mitigated in shMst1/2 knockdown cells (Fig. 1c, d). Furthermore, electron microscopic analysis revealed that the increase in the number of autophagosomes upon CCCP and DFP treatment was significantly reduced in shMst1/2 cells (Fig. 1e). Impaired CCCP- and DFP-induced mitophagy under Mst1/2 knockdown conditions was also observed in HeLa cells expressing Parkin (Supplementary Fig. 1b–d). Moreover, Mst1 and Mst2 were double knocked down using another set of shRNAs (shMst1/2 #2) or employing small interfering RNAs (siMst1/2) targeting different sites on the Mst1 and Mst2 genes, which similarly reduced mitophagy induction in response to CCCP and DFP treatments (Supplementary Fig. 1e–j). These results collectively suggest that Mst1/2 are required for the induction of mitophagy upon mitochondrial stress.

### Kinase activity of Mst1/2 is required for the induction of mitophagy upon mitochondrial stress

**Fig. 2:**
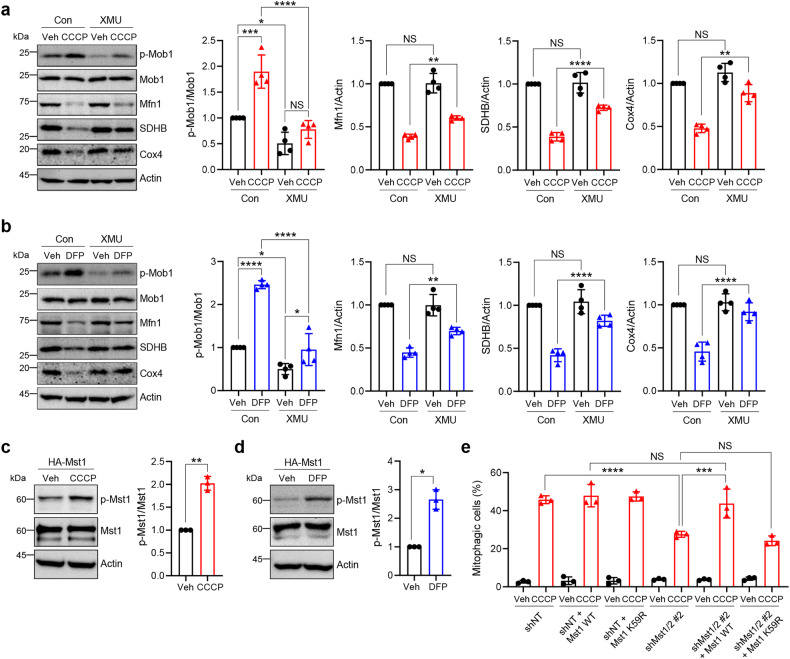
Critical role of Mst1/2 kinase activity in mitophagy induction in response to mitochondrial stress. HEK293 cells were pretreated with XMU-MP-1 (5 μM) for 16 h and then cotreated with CCCP (10 μM) (**a**) or DFP (1 mM) (**b**) in the presence of XMU-MP-1 for 24 h. Western blotting analysis was performed using the indicated antibodies. The results from four biological replicates are shown on the right as the mean ± SD. HEK293 cells were transfected with the HA-Mst1 expression plasmid and treated with CCCP (10 μM) (**c**) or DFP (1 mM) (**d**) for 24 h. Western blotting analysis was performed using the indicated antibodies. The results from three biological replicates are shown on the right as the mean ± SD. **e** HEK293 cells expressing mt-Keima and either control nontargeting shRNA (shNT) or Mst1 and Mst2 shRNAs (shMst1/2 #2) were transfected with Mst1 WT or Mst1 K59R expression plasmids. After treatment with CCCP (10 μM) for 6 h, mitophagy was analyzed via flow cytometry. The results from three biological replicates are shown as the mean ± SD. Significance was determined by two-way ANOVA (**a**, **b**, **e**) with Šidák’s multiple-comparison test or Student’s *t* test (**c**, **d**). *P < 0.05; **P < 0.01; ***P < 0.001; ****P < 0.0001. NS not significant.

Mst1/2 mainly function through their kinase activity^14,39^. To further verify the role of Mst1/2 in mitophagy induction upon mitochondrial stress, we next examined the effect of XMU-MP-1, a potent ATP-competitive inhibitor of both Mst1 and Mst2^40^. As shown in Fig. 2a, b, CCCP and DFP treatment resulted in an increase in the level of phosphorylated MOB1, a representative downstream target of Mst1/2^39^, suggesting the induction of Mst1/2 kinase activity. Interestingly, XMU-MP-1 treatment effectively inhibited the CCCP- and DFP-induced downregulation of Mfn1, SDHB and Cox4, suggesting the suppression of mitophagy induction.

Western blot analysis revealed that the level of Thr183 phosphorylation, an indicator of Mst1 activation^39^, was significantly increased upon CCCP and DFP treatment (Fig. 2c, d), suggesting that Mst1 is activated upon mitochondrial stress. To verify the necessity of Mst1/2 kinase activity for mitophagy induction, we exogenously expressed either wild-type Mst1 or the kinase-dead form of Mst1 (Mst1 K59R)^25^ in Mst1/2 double-knockdown cells (Supplementary Fig. 2a). While wild-type Mst1 significantly restored CCCP-induced mitophagy, kinase-dead Mst1 did not significantly rescue mitophagy induction upon CCCP treatment (Fig. 2e). Exogenous expression of wild-type Mst2 or the kinase-dead form of Mst2 (Mst2 K56R)^26,27^ also had similar effects on CCCP-induced mitophagy (Supplementary Fig. 2b, c). These results suggest that Mst1/2 regulate mitophagy induction upon mitochondrial stress through its kinase activity.

### Mst1/2-induced mitophagy upon mitochondrial stress is independent of the PINK1-Parkin pathway and the canonical Hippo pathway

**Fig. 3:**
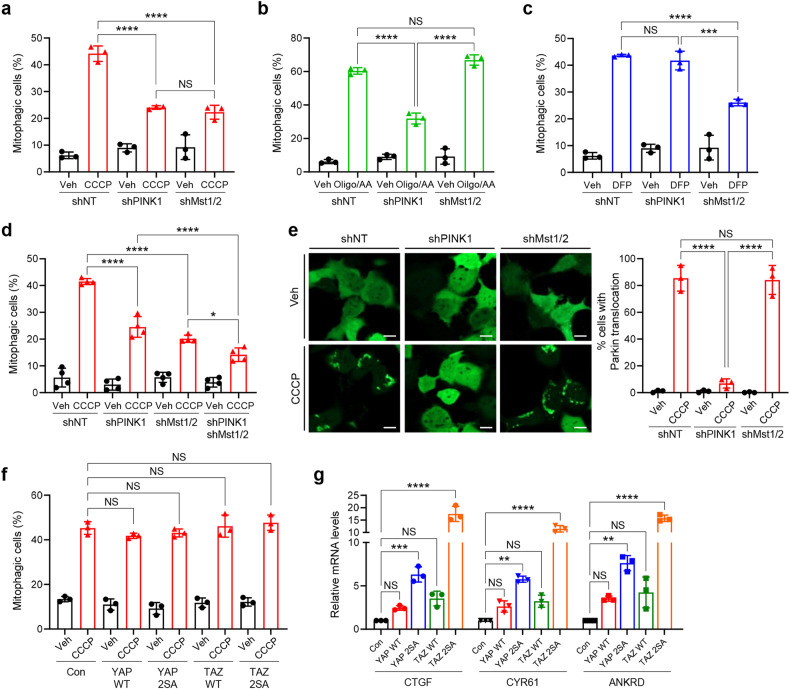
Mst1/2 induces mitophagy upon mitochondrial stress independent of PINK1-Parkin and the canonical Hippo pathway. HEK293 cells expressing mt-Keima and either control nontargeting shRNA (shNT), PINK1 shRNA (shPINK1) or Mst1 and Mst2 shRNAs (shMst1/2) were treated with CCCP (10 μM) for 6 h (**a**), Oligo/AA (25 nM/250 nM) for 24 h (**b**) or DFP (1 mM) for 24 h (**c**), and mitophagy levels were analyzed by flow cytometry. The results from three biological replicates are shown as the mean ± SD. **d** The three cell lines in (**a**) and HEK293 cells expressing mt-Keima, PINK1 shRNA (shPINK1), and Mst1 and Mst2 shRNAs (shMst1/2) were treated with CCCP (10 μM) for 6 h, and mitophagy levels were analyzed by flow cytometry. The results from four biological replicates are shown as the mean ± SD. **e** HEK293 cells expressing control nontargeting shRNA (shNT), PINK1 shRNA (shPINK1) or Mst1/2 shRNA (shMst1/2) were transfected with the YFP-Parkin plasmid. After treatment with CCCP (10 μM) for 2 h, the translocation of YFP-Parkin into mitochondria was analyzed via confocal microscopy. The results from three biological replicates are shown as the mean ± SD. **f**–**g** HEK293 cells expressing mt-Keima and either YAP WT, TAP 2SA, TAZ WT or TAZ 2SA were treated with CCCP (10 μM) for 6 h, after which mitophagy levels were analyzed via flow cytometry (**f**). Total RNA samples were harvested after 2 days of YAP WT, TAP 2SA, TAZ WT or TAZ 2SA expression, and the levels of CTGF, CYR61 or ANKRD mRNA were analyzed by real-time PCR (**g**). The results from three biological replicates are shown as the mean ± SD. Significance was determined by two-way ANOVA with Šidák’s multiple-comparison test. *P < 0.05; **P < 0.01; ***P < 0.001; ****P < 0.0001. NS not significant.

To understand the molecular mechanism of Mst1/2-mediated mitophagy induction, we examined the interaction of Mst1/2 with the PINK1-Parkin pathway, the representative mitophagy pathway, in response to mitochondrial stress^9,11^. We first compared the effects of PINK1 or Mst1/2 knockdown on mitophagy induction under various mitochondrial stress conditions. PINK1 plays a critical role in mitophagy induction upon treatment with CCCP or a combination of oligomycin and antimycin A, whereas PINK1 is not essential for DFP-induced mitophagy^38^. Consistently, we found that knockdown of PINK1 (Supplementary Fig. 3a) significantly suppressed CCCP- and oligomycin/antimycin A-induced mitophagy, while DFP-induced mitophagy did not change upon PINK1 knockdown (Fig. 3a–c). Interestingly, knockdown of Mst1/2 suppressed CCCP- and DFP-induced mitophagy, but oligomycin/antimycin A-induced mitophagy was not affected (Fig. 3a–c). These results suggest that the Mst1/2 and PINK1-Parkin pathways may regulate mitophagy induction independently of the type of mitochondrial stress. Consistent with this notion, CCCP-induced mitophagy was further suppressed after PINK1 and Mst1/2 knockdown compared with after PINK1 knockdown or Mst1/2 knockdown alone (Fig. 3d). Moreover, Parkin translocation into mitochondria was not affected by knockdown of Mst1/2, while PINK1 knockdown completely abolished Parkin translocation (Fig. 3e). These results further support that PINK1 and Mst1/2 independently regulate mitophagy induction upon CCCP treatment. In addition, knockdown of both PINK1 and Mst1/2 in other cell lines, including HeLa cells expressing Parkin and SH-SY5Y cells, also resulted in a significant decrease in CCCP-induced mitophagy (Supplementary Fig. 3b, c), further confirming that both PINK1 and Mst1/2 play a critical role in CCCP-induced mitophagy, although the exact proportions of mitophagy attributable to each pathway are difficult to determine.

Since Mst1/2 regulate many cellular processes mainly through the negative regulation of two transcription coactivators, YAP and TAZ, in the canonical Hippo pathway^15,39^, we next examined the effect of YAP and TAZ ectopic overexpression on mitophagy induction upon mitochondrial stress. Overexpression of the wild-type or active forms of YAP and TAZ (YAP 2SA and TAZ 2SA, respectively) had no effect on mitophagy induction upon CCCP treatment (Fig. 3f), whereas overexpression of the wild-type or active forms of YAP and TAZ markedly induced the expression of their target genes, including CTGF, CYF61, and ANKRD (Fig. 3g). These results suggest that Mst1/2 may not regulate mitophagy induction through the canonical Hippo pathway.

### Mst1/2 modulate mitophagy induction upon mitochondrial stress through BNIP3

**Fig. 4:**
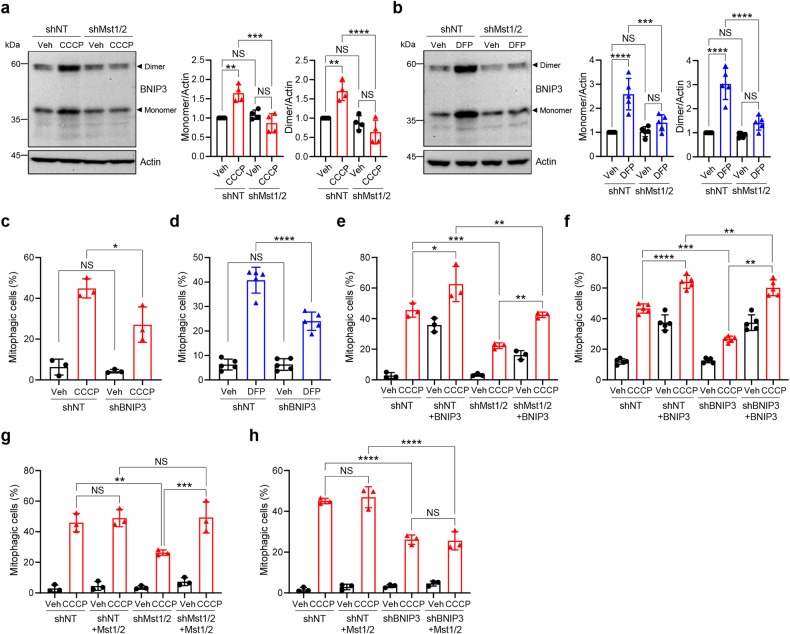
BNIP3 is required for Mst1/2-mediated mitophagy induction upon mitochondrial stress. HEK293 cells expressing either control nontargeting shRNA (shNT) or Mst1 and Mst2 shRNAs (shMst1/2) were transfected with the BNIP3 expression plasmid. After treatment with CCCP (10 μM) (**a**) or DFP (1 mM) (**b**) for 24 h, western blotting analysis was performed using the indicated antibodies. The results from four or five biological replicates are shown on the right as the mean ± SD. HEK293T cells expressing mt-Keima and either control nontargeting shRNA (shNT) or BNIP3 shRNA (shBNIP3) were treated with CCCP (10 μM) for 6 h (**c**) or DFP (1 mM) for 24 h (**d**), and mitophagy levels were analyzed by flow cytometry. The results from three or five biological replicates are shown as the mean ± SD. HEK293 cells expressing mt-Keima and either control nontargeting shRNA (shNT), Mst1 and Mst2 shRNAs (shMst1/2) or BNIP3 shRNA (shBNIP3) were transfected with BNIP3 (**e**, **f**) or Mst1 and Mst2 (**g**, **h**) expression plasmids. Cells were treated with CCCP (10 μM) for 6 h, after which mitophagy levels were analyzed via flow cytometry. The results from three or five biological replicates are shown as the mean ± SD. Significance was determined by two-way ANOVA with Šidák’s multiple-comparison test. *P < 0.05; **P < 0.01; ***P < 0.001; ****P < 0.0001. NS not significant.

We recently revealed that Mst1/2 regulate mitophagy through the well-known mitophagy receptor BNIP3 during the metabolic remodeling of adipocytes^41^. Mst1/2-induced phosphorylation of BNIP3 enhances BNIP3 stability, resulting in elevated intracellular levels of BNIP3 and mitophagy induction. To test whether BNIP3 is also involved in mitophagy induction upon mitochondrial stress, we first examined the level of BNIP3 upon CCCP and DFP treatment and the effect of Mst1/2 knockdown on BNIP3 levels. As shown in Fig. 4a, upon CCCP treatment, the levels of both the monomeric and dimeric forms of ectopically expressed BNIP3 increased approximately 1.6-fold and 1.7-fold, respectively. However, this increase was completely abrogated by the double knockdown of Mst1/2 (Fig. 4a). DFP treatment also increased the level of monomeric and dimeric BNIP3 by approximately 2.6-fold and 3.0-fold, respectively, and these increases were abrogated by the double knockdown of Mst1/2 (Fig. 4b), suggesting that Mst1/2 are required for the stabilization of BNIP3 upon mitochondrial stress. Moreover, knockdown of BNIP3 suppressed mitophagy induction upon treatment with CCCP or DFP by approximately 40% and 42%, respectively (Fig. 4c, d; Supplementary Fig. 4a). These results further support that BNIP3 is required for the induction of mitophagy upon CCCP and DFP treatment.

To verify that Mst1/2 regulate mitophagy induction upon mitochondrial stress through BNIP3, we then performed a series of rescue experiments. To restore Mst1 expression, we generated a nondegradable Mst1 variant by introducing mutations within the shRNA target site, while for Mst2 and BNIP3 reintroduction, we utilized wild-type Mst2 and BNIP3 expression vectors, as Mst2 and BNIP3 shRNAs target the 3′-UTRs of their respective genes. The reintroduction of BNIP3 successfully reinstated mitophagy induction upon CCCP treatment in both Mst1/2 double-knockdown and BNIP3-knockdown cells (Fig. 4e, f; Supplementary Fig. 4b, c). Conversely, while Mst1/2 reintroduction rescued mitophagy induction in Mst1/2 double knockdown cells, it was unable to restore mitophagy induction in BNIP3 knockdown cells (Fig. 4g, h; Supplementary Fig. 4d, e). These results suggest that Mst1/2 function as upstream regulators of BNIP3 in mediating mitophagy induction upon CCCP treatment. In addition, Mst1/2 reintroduction restored BNIP3 stabilization upon CCCP treatment in Mst1/2 double-knockdown cells (Supplementary Fig. 4f), further confirming that Mst1/2 are responsible for BNIP3 stabilization.

### Mst1/2 play critical roles in maintaining mitochondrial homeostasis and survival under neurotoxic conditions

**Fig. 5:**
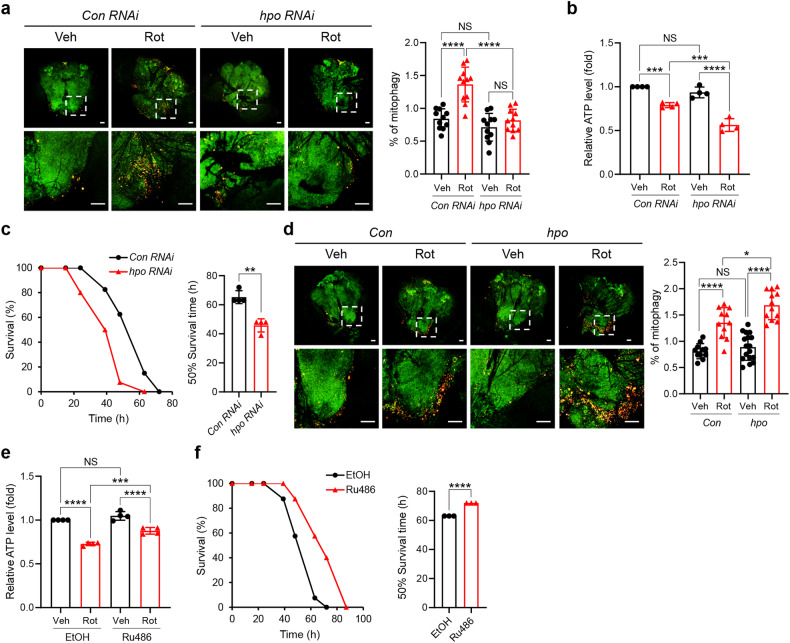
Essential role of Mst1/2 in the response to mitochondrial stress in *Drosophila*. **a**, **b** Seven-day-old *Drosophila* expressing mt-Keima and either *control RNAi* (*appl>mt-Keima, control RNAi; Con RNAi*) or *hippo RNAi* (*appl>mt-Keima, hpo RNAi; hpo RNAi*) were treated with DMSO (Veh) or rotenone (Rot, 7.5 mM) for 24 h, after which mitophagy levels in the brain were determined via confocal microscopy (**a**). Scale bars: 50 μm (upper). Scale bars: 50 μm (bottom). The quantified mitophagy levels are shown on the right as the mean ± SD (*n* ≧ 10 per sample). ATP levels in the brain were assessed, and the results from four biological replicates are shown as the mean ± SD (**b**). **c** Seven-day-old *Drosophila* expressing either *control RNAi* (*Con RNAi*) or *hippo RNAi* (*hpo RNAi*) were treated with rotenone (Rot, 7.5 mM), after which survival was assessed. The quantified 50% survival time from four biological replicates is shown on the right as the mean ± SD. **d** Seven-day-old *Drosophila* expressing mt-Keima (Con) alone or in combination with *hippo* (*hpo*) were treated with DMSO (Veh) or rotenone (Rot, 7.5 mM) for 24 h, and mitophagy levels in the brain were analyzed via confocal microscopy. Scale bars: 50 μm (upper). Scale bars: 50 μm (bottom). The quantified mitophagy levels are shown on the right as the mean ± SD (*n* ≧ 10 per sample). **e**, **f** Seven-day-old *Drosophila* (*elav-GS>hpo)* were treated with either ethanol (EtOH) or Ru486 (10 μM) for 3 days to induce *hippo* expression. Rotenone (7.5 mM) was administered for 24 h, after which ATP levels in the brain were assessed (**e**). The results from three biological replicates are shown as the mean ± SD. Rotenone (7.5 mM) was administered, after which cell survival was assessed (**f**). The quantified 50% survival time from three biological replicates is shown on the right as the mean ± SD. Significance was determined by two-way ANOVA (**a**, **b**, **d**, **e**) with Šidák’s multiple-comparison test or Student’s *t* test (**c**, **f**). *P < 0.05; **P < 0.01; ***P < 0.001; ****P < 0.0001. NS not significant.

To investigate the physiological relevance of Mst1/2-mediated mitophagy induction, we next examined the role of Mst1/2 in response to rotenone, a well-known neurotoxin that induces mitochondrial dysfunction by inhibiting complex I, in a *Drosophila* model. Confocal microscopy analysis of mt-Keima fluorescence revealed a significant increase (approximately 1.6-fold) in brain mitophagy levels upon rotenone treatment (Fig. 5a). However, neuron-specific knockdown of *hippo* (*hpo*) using the *appl-Gal4* driver, the *Drosophila* ortholog of Mst1/2, abrogated mitophagy induction upon rotenone treatment (Fig. 5a), suggesting that *hpo* is required for mitophagy induction in response to rotenone. Furthermore, *hpo* knockdown resulted in further decreases in ATP levels (approximately 18%) and *Drosophila* survival (approximately 30%) upon rotenone treatment (Fig. 5b, c; Supplementary Fig. 5a). Conversely, neuron-specific expression of *hpo* led to a significant increase in mitophagy (approximately 25%) upon rotenone treatment (Fig. 5d). Simultaneously, inducing adult-specific *hpo* expression via *elav-GS-Gal4* activation via the RU486 compound ameliorated the decrease in ATP levels and improved survival upon rotenone treatment (Fig. 5e, f; Supplementary Fig. 5b). Interestingly, dopaminergic neuron-specific expression of *hpo* via *TH-Gal4* also ameliorated the loss of dopaminergic neurons upon rotenone treatment (Supplementary Fig. 5c). These results suggest that *hpo* is important not only for mitophagy induction but also for maintaining mitochondrial homeostasis and organismal viability under neurotoxic conditions in *Drosophila*.

**Fig. 6:**
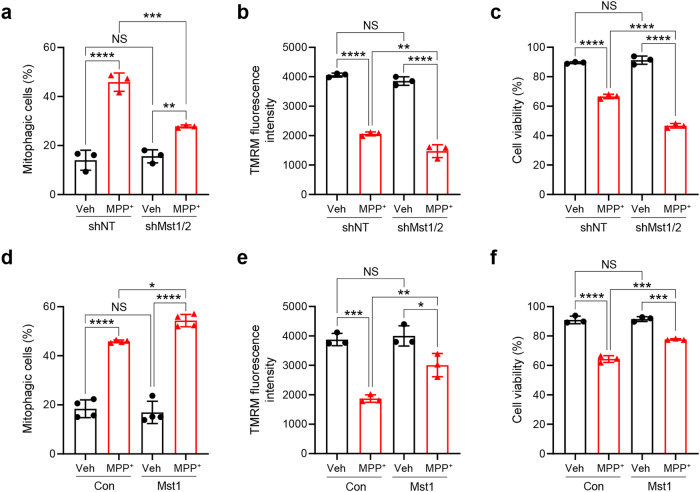
Essential role of Mst1/2 in response to mitochondrial stress in neuronal cells. **a**–**c** SH-SY5Y cells expressing either control nontargeting shRNA (shNT) or Mst1 and Mst2 shRNAs (shMst1/2) were treated with MPP^+^ (500 μM) for 24 h, and mitophagy levels were analyzed via flow cytometry (**a**). The mitochondrial membrane potential was assessed by TMRM staining (**b**), and cell viability was analyzed (**c**). The results from three biological replicates are shown as the mean ± SD. **d**–**f**) SH-SY5Y cells were transfected with either vector (Con) or the Mst1 expression plasmid and treated with MPP^+^ (500 μM) for 24 h. Mitophagy levels were analyzed via flow cytometry (**d**). The mitochondrial membrane potential was analyzed by TMRM staining (**e**), and cell viability was analyzed (**f**). The results from at least three biological replicates are shown as the mean ± SD. Significance was determined by two-way ANOVA with Šidák’s multiple-comparison test. *P < 0.05; **P < 0.01; ***P < 0.001; ****P < 0.0001. NS not significant.

To verify the role of Mst1/2 in response to neurotoxic conditions, we next examined the effect of Mst1/2 knockdown in SH-SY5Y human neuroblastoma cells treated with MPP^+^, a well-known neurotoxin that induces mitochondrial dysfunction by inhibiting complex I. Similar to the findings in *Drosophila*, Mst1/2 double knockdown in SH-SY5Y cells significantly reduced mitophagy induction (approximately 40%) and further decreased mitochondrial membrane potential and cell viability upon MPP^+^ treatment (approximately 13% and 13%, respectively) (Fig. 6a–c, Supplementary Fig. 6a). Conversely, ectopic expression of Mst1 further increased mitophagy levels by approximately 18% and ameliorated the decreases in mitochondrial membrane potential and cell viability caused by MPP^+^ treatment by approximately 27% and 20%, respectively (Fig. 6d–f, Supplementary Fig. 6b). Ectopic expression of Mst2 also had similar effects on mitophagy induction, the mitochondrial membrane potential, and cell viability upon MPP^+^ treatment (Supplementary Fig. 6c–f). These results suggest that Mst1/2 play important roles in mitophagy induction and in the potential maintenance of mitochondrial homeostasis and viability under neurotoxic conditions.

### Mst1 ameliorates MPTP-induced mitochondrial dysfunction and dopaminergic neuronal death in mice

**Fig. 7:**
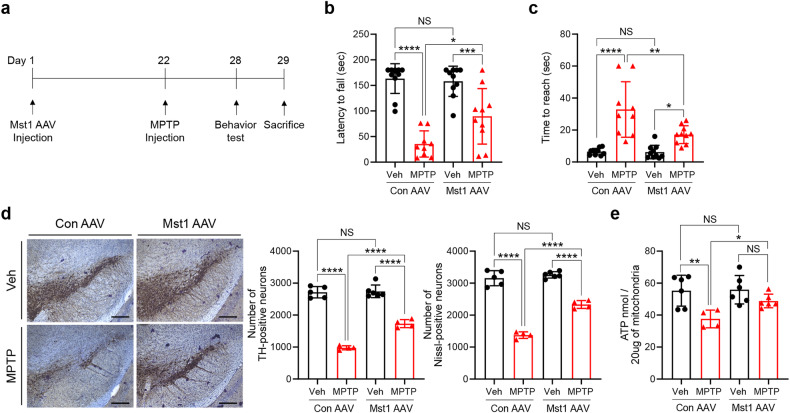
Mst1 ameliorates MPTP-induced mitochondrial dysfunction and neuronal cell death. **a** Schematic diagram of the animal experiments. **b**–**e** C57BL/6 J mice (8 weeks old) were injected with either control AAV (Con AAV) or Mst1 AAV into the substantia nigra (SN) of the right hemisphere. Four weeks after AAV injection, the latency to fall in the rotarod test (**b**) and the time to reach the bottom in the pole test (**c**) were measured (Con AAV + Veh, *n* = 11; Con AAV + MPTP, *n* = 9; Mst1 AAV + Veh, *n* = 10; Mst1 AAV + MPTP, *n* = 10). The mice in (**b**, **c**) were sacrificed, and TH-positive neurons in the SN were analyzed (**d**). Unbiased stereology counts of TH^+^ neurons and Nissl-stained neurons are shown on the right as the mean ± SD (Con AAV + Veh, *n* = 5; Con AAV + MPTP, *n* = 4; Mst1 AAV + Veh, *n* = 4; Mst1 AAV + MPTP, *n* = 4). Scale bars: 0.2 mm. Mitochondria were isolated from the SN, and ATP levels were analyzed (**e**) (Con AAV + Veh, *n* = 6; Con AAV + MPTP, *n* = 4; Mst1 AAV + Veh, *n* = 6; Mst1 AAV + MPTP, *n* = 6). Scale bars: 20 μm. Significance was determined by two-way ANOVA with Šidák’s multiple-comparison test. *P < 0.05; **P < 0.01; ***P < 0.001; ****P < 0.0001. NS not significant.

Our findings in *Drosophila* and SH-SY5Y cells suggest that Mst1 or Mst2 expression may protect neuronal cells against neurotoxic treatments. To further validate the effect of Mst1/2 expression against neurotoxic treatments under physiological conditions, we examined the effect of AAV-mediated Mst1 expression in the mouse SN using the MPTP mouse model, a well-established model of Parkinson’s disease^42^. We injected the Mst1 AAV virus into the SN three weeks prior to MPTP treatment (Fig. 7a). Immunofluorescence staining performed four weeks after stereotactic injection of Mst1 AAV confirmed the expression of Mst1 in TH-positive neurons (Supplementary Fig. 7). Interestingly, Mst1 AAV injection ameliorated the MPTP-induced reduction in latency to fall on the rotarod test by approximately 24% (Fig. 7b). Additionally, the time taken to reach the floor (time to reach) in the pole test was improved by approximately 55% following Mst1 AAV injection (Fig. 7c), suggesting that Mst1 expression protects neurons in the SN against MPTP-induced toxicity. Consistently, unbiased stereological counting of TH-positive neurons in the SN revealed that the reduction in TH-positive neurons upon MPTP injection was ameliorated by approximately 24% by Mst1 AAV injection (Fig. 7d). Moreover, analysis of ATP levels in mitochondria isolated from the SN revealed that Mst1-AAV injection significantly mitigated the MPTP-mediated decrease in ATP levels by approximately 19% (Fig. 7e). These results suggest that Mst1 expression alleviates behavioral impairments and enhances the viability of TH-positive neurons in MPTP-induced Parkinson’s disease model mice.

## Discussion

Mitophagy induction upon mitochondrial stress has been shown to be critical for maintaining mitochondrial homeostasis and cellular viability, particularly in neuronal cells^43,44^. In this study, we found that Mst1/2 are indispensable for the proper induction of mitophagy in response to various mitochondrial stresses. Mitophagy induction upon CCCP and DFP treatment was significantly decreased in Mst1/2 double-knockdown cells. The ATP-competitive Mst1/2 inhibitor XMU-MP-1 also significantly inhibited mitophagy induction upon CCCP and DFP treatment. Moreover, neuron-specific knockdown of *hippo* significantly diminished mitophagy induction upon exposure to rotenone in a *Drosophila* model. A similar reduction in mitophagy levels upon MPP^+^ treatment was also observed in SH-SY5Y human neuroblastoma cells. These results indicate that Mst1/2 are essential for the proper induction of mitophagy under various mitochondrial stresses. Recently, we reported that Mst1/2 regulate adipocyte mitophagy during metabolic remodeling^41^. However, the involvement of Mst1/2 in mitophagy induction upon mitochondrial stress has not been studied. Our study revealed for the first time the critical role of Mst1/2 in mitophagy induction under mitochondrial stress.

The physiological importance of Mst1/2-mediated mitophagy is underscored by our observations in both *Drosophila* and SH-SY5Y cells under neurotoxic conditions. Neuron-specific knockdown of Mst1/2 significantly diminished mitophagy induction and exacerbated the reduction in ATP levels, mitochondrial membrane potential and cellular viability upon exposure to neurotoxins, highlighting the importance of Mst1/2 in maintaining mitochondrial homeostasis and promoting cellular survival under conditions of stress. Recent extensive studies have demonstrated that defective mitophagy is closely associated with mitochondrial dysfunction and the pathogenesis of various neurodegenerative diseases, including Alzheimer’s disease and Parkinson’s disease^45^. In addition, stimulating mitophagy has recently been shown to have beneficial effects on various animal models of neurodegenerative disease^46–49,45^. Consistent with this notion, AAV-mediated Mst1 expression in the mouse SN ameliorated the pathological phenotypes of MPTP-induced Parkinson’s disease model mice. These findings are further corroborated by our experiments involving AAV-mediated Mst1 expression in a mouse model of Parkinson’s disease, in which Mst1 expression ameliorated motor deficits and improved the survival of dopaminergic neurons. Thus, in addition to the critical role of Mst1 and Mst2 in organ size control^15,16^, our results underscore the important role of Mst1/2 in maintaining mitochondrial health and protecting against neurodegenerative insults.

We found that Mst1/2 kinase activity is necessary for mitophagy induction upon mitochondrial stress. The phosphorylation of Thr183 indicated Mst1 activation upon CCCP and DFP treatment. Moreover, the Mst1/2 inhibitor XMU-MP-1 suppressed CCCP- and DFP-induced mitophagy. The rescue experiment results using wild-type and kinase-dead Mst1 and Mst2 further support the involvement of Mst1/2 kinase activity in mitophagy induction upon mitochondrial stress. Mechanistic analysis revealed that the mitophagy receptor BNIP3 is required for Mst1/2-mediated mitophagy induction. Previous studies have shown that BNIP3 plays a critical role in mitophagy induction in response to various cellular stresses, including hypoxia^50,51^. We observed in this study that knockdown of Mst1/2 suppressed the increase in BNIP3 levels upon CCCP and DFP treatment, suggesting that Mst1/2 are required for the stabilization of BNIP3 upon mitochondrial stress. Consistent with our observation, induction of BNIP3 upon DFP treatment has also been observed in various recent studies^52–54^. Moreover, the essential role of BNIP3 in DFP-induced mitophagy has been observed in SH-SY5Y cells^52^. In addition, suppression of CCCP-induced mitophagy upon BNIP3 knockdown has also been observed in A549 cells^55^. Thus, studies from our group and others suggest that BNIP3 plays a pivotal role in mitophagy induction under mitochondrial stress conditions. Although our rescue experiment results suggest that BNIP3 is a critical downstream mediator of Mst1/2 during mitophagy upon mitochondrial stress, whether Mst1/2 directly phosphorylates the BNIP3 Thr88 residue, as we observed in a previous study^41^, should be further verified in future studies. In addition, given that Mst1/2 regulate various target proteins through its kinase activity^14,39^, whether Mst1/2 regulate mitophagy signaling pathways through their phosphorylation needs to be investigated.

Interestingly, we found that Mst1/2-mediated mitophagy induction operates independently of the well-established PINK1-Parkin pathway and the canonical Hippo pathway. While PINK1 plays a pivotal role in CCCP- and oligomycin/antimycin A-induced mitophagy, Mst1/2 regulate mitophagy induction in response to CCCP and DFP treatment, indicating a distinct mode of action. Furthermore, the absence of an effect on mitophagy induction upon overexpression of YAP and TAZ suggested that Mst1/2 modulates mitophagy through a pathway distinct from the canonical Hippo–YAP/TAZ axis in response to mitochondrial stress. These results underscore the complexity of mitophagy regulation and highlight the need for further exploration to elucidate the specific molecular pathways through which Mst1/2 exert their effects. Although our results suggest that Mst1/2 regulates mitophagy induction independently of the PINK1-Parkin pathway, the interaction between the Mst1/2-BNIP3 axis and the PINK1-Parkin pathway needs to be investigated in more detail. In particular, although our results demonstrated significant reductions in mitophagy upon PINK1 and Mst1/2 knockdown, precisely estimating the proportional contributions of each pathway remains challenging, underscoring the need for additional sophisticated experiments in future studies. Furthermore, considering that Lats kinase is a well-known downstream target of Mst1/2 in the Hippo pathway, it is imperative to investigate the potential involvement of Lats kinase in the induction of mitophagy in response to mitochondrial stresses. Notably, several studies have previously shown the inhibitory effect of Mst1 on mitophagy in various disease models, such as cardiac ischemia‒reperfusion models and nonalcoholic fatty liver disease (NAFLD) models^56–58^. These results suggest that the complex and pleiotropic role of Mst1/2 in mitophagy regulation depends on the cellular context and stimulus. The differential mechanisms through which Mst1/2 modulate mitophagy in various contexts should be explored in additional studies. Interestingly, our findings in both *Drosophila* and the MPTP mouse model also suggest that Mst1/2 could be potential targets for Parkinson’s disease treatment. However, the therapeutic relevance of Mst1/2 in Parkinson’s disease requires careful validation. We are currently conducting an in-depth investigation into the potential pathogenic link between Mst1/2 and Parkinson’s disease, including examination of the expression and genetic alterations of Mst1/2 in Parkinson’s disease patients and relevant models.

In conclusion, our study reveals a novel regulatory axis involving Mst1/2 in mitophagy induction upon mitochondrial stress. Our results suggest that Mst1/2 are essential for mitophagy induction in response to mitochondrial stress, acting through their kinase activity and BNIP3. Furthermore, our findings suggest that Mst1/2 play a crucial role in maintaining mitochondrial homeostasis and cellular survival under neurotoxic conditions. Although the specific molecular mechanisms through which Mst1/2 regulate BNIP3 stability and crosstalk with other signaling pathways need to be further investigated, our study provides insights into the potential therapeutic relevance of targeting the Mst1/2 pathway to enhance mitochondrial health and protect against neurodegenerative insults.

### Supplementary information

Supplementary Figures
