# Supplementary material for: The Mst1/2-BNIP3 axis is required for mitophagy induction and neuronal viability under mitochondrial stress
Source: Exp Mol Med. 2024 Mar 5;56(3):674–85. doi: 10.1038/s12276-024-01198-y (PMC10984967; doi:10.1038/s12276-024-01198-y)
Supplement: Supplementary file 1 — Supplementary Figures [file 12276_2024_1198_MOESM1_ESM.pdf]

Supplementary materials

The Mst1/2-BNIP3 axis is required for mitophagy induction and neuronal viability under mitochondrial stress

Dae Jin Jeong[1,2,6], Jee-Hyun Um[1,2,6], Young Yeon Kim[1,2,6], Dong Jin Shin[1,2], Sangwoo Im[1,2], Kang-Min Lee[1,2], Yun-Hee Lee[3], Dae-sik Lim[4], Donghoon Kim[2,5], and Jeanho Yun[1,2,*]

[1] Department of Biochemistry, College of Medicine, Dong-A University, Busan, Republic of Korea

[2] Department of Translational Biomedical Sciences, Graduate School of Dong-A University, Busan, Republic of Korea

[3] College of Pharmacy, Research Institute of Pharmaceutical Sciences, Seoul National University, Seoul, Republic of Korea

[4] Department of Biological Sciences, National Creative Research Center for Cell Plasticity, Korea Advanced Institute of Science and Technology (KAIST), Daejeon, South Korea.

[5] Department of Pharmacology, College of Medicine, Dong-A University, Busan, Korea.

[6] These authors contributed equally: Dae Jin Jeong, Jee-Hyun Um, Young Yeon Kim

*email: yunj@dau.ac.kr

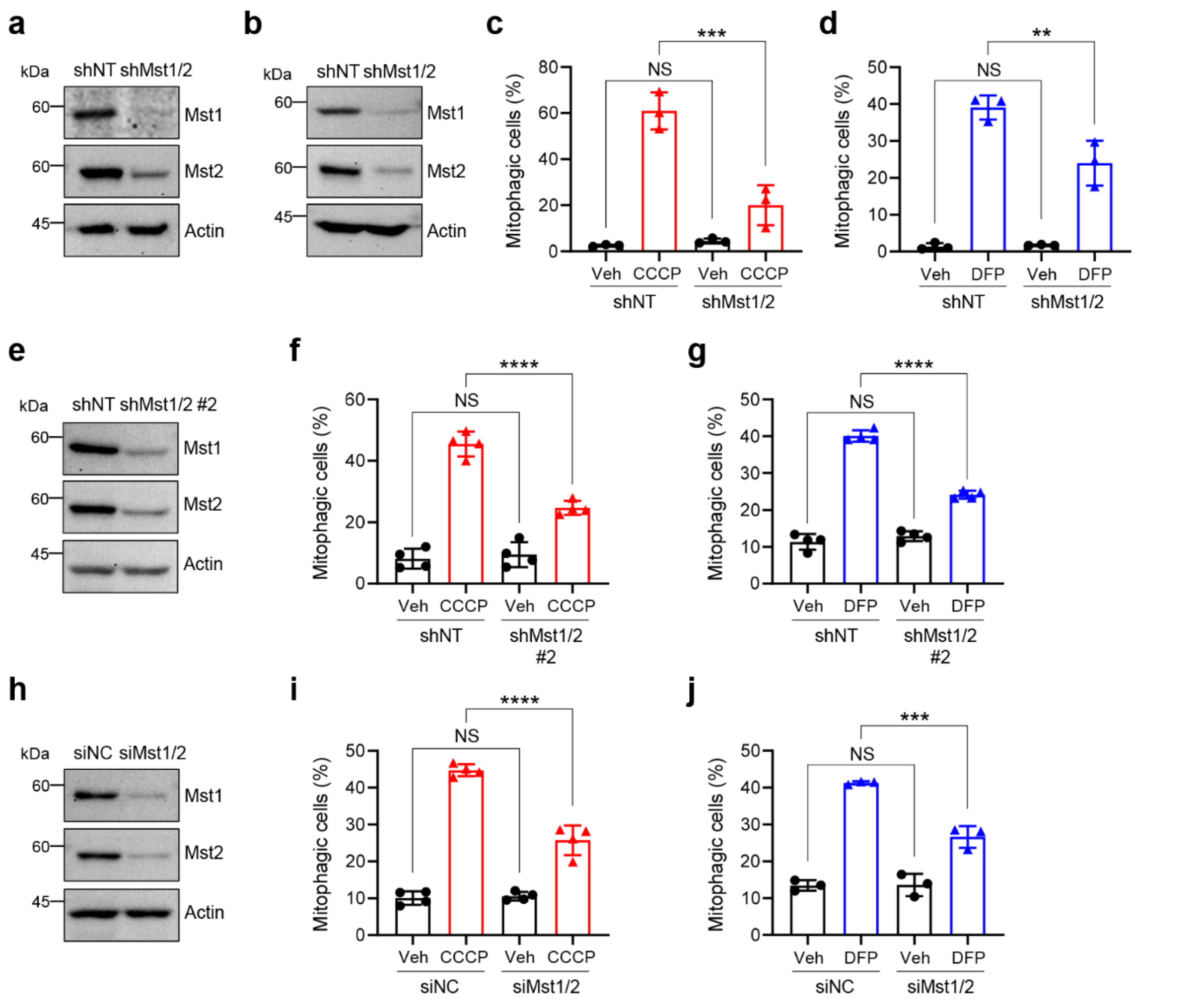

Supplementary Figure 1. Verification of the role of Mst1/2 in mitophagy induction upon mitochondrial stress.

(a-b) HEK293 (a) or HeLa-Parkin cells (b) expressing mt-Keima and either control nontargeting shRNA (shNT) or Mst1 and Mst2 shRNAs (shMst1/2) were subjected to western blot analysis using the indicated antibodies. (c-d) HeLa-Parkin cells expressing mt-Keima and either control nontargeting shRNA (shNT) or Mst1 and Mst2 shRNAs (shMst1/2) were treated with CCCP (10 μM) for 6 h (c) or treated with DFP (1 mM) for 24 h (d). Mitophagy levels were analyzed by flow cytometry, and the results from three biological replicates are shown on the right as the mean ± SD. (e-g) HEK293 cells expressing mt-Keima and either control nontargeting shRNA (shNT) or Mst1 and Mst2 shRNAs (shMst1/2 #2) were subjected to western blot analysis using the indicated antibodies (e). HEK293 cells expressing mt-Keima and either control nontargeting shRNA (shNT) or Mst1 and Mst2 shRNAs (shMst1/2 #2) were treated with CCCP (10 μM) for 6 h (f) or DFP (1 mM) for 24 h (g), and mitophagy levels were analyzed via mt-Keima based flow cytometry. The results from four biological replicates are

shown as the mean ± SD. (h-i) HEK293 cells expressing mt-Keima were transfected with either negative control siRNA (siNC) or Mst1 and Mst2 siRNAs (siMst1/2) and subjected to western blot analysis using the indicated antibodies (h). HEK293 cells expressing mt-Keima were transfected with either negative control siRNA (siNC) or Mst1 and Mst2 siRNAs (siMst1/2) and treated with CCCP (10 μM) for 6 h (i) or DFP (1 mM) for 24 h (j), and mitophagy levels were analyzed via mt-Keima based flow cytometry. The results from four biological replicates are shown as the mean ± SD. Significance was determined by two-way ANOVA with Šidák's multiple-comparison test. $^{**}P < 0.01$; $^{***}P < 0.001$; $^{****}P < 0.0001$. NS, not significant.

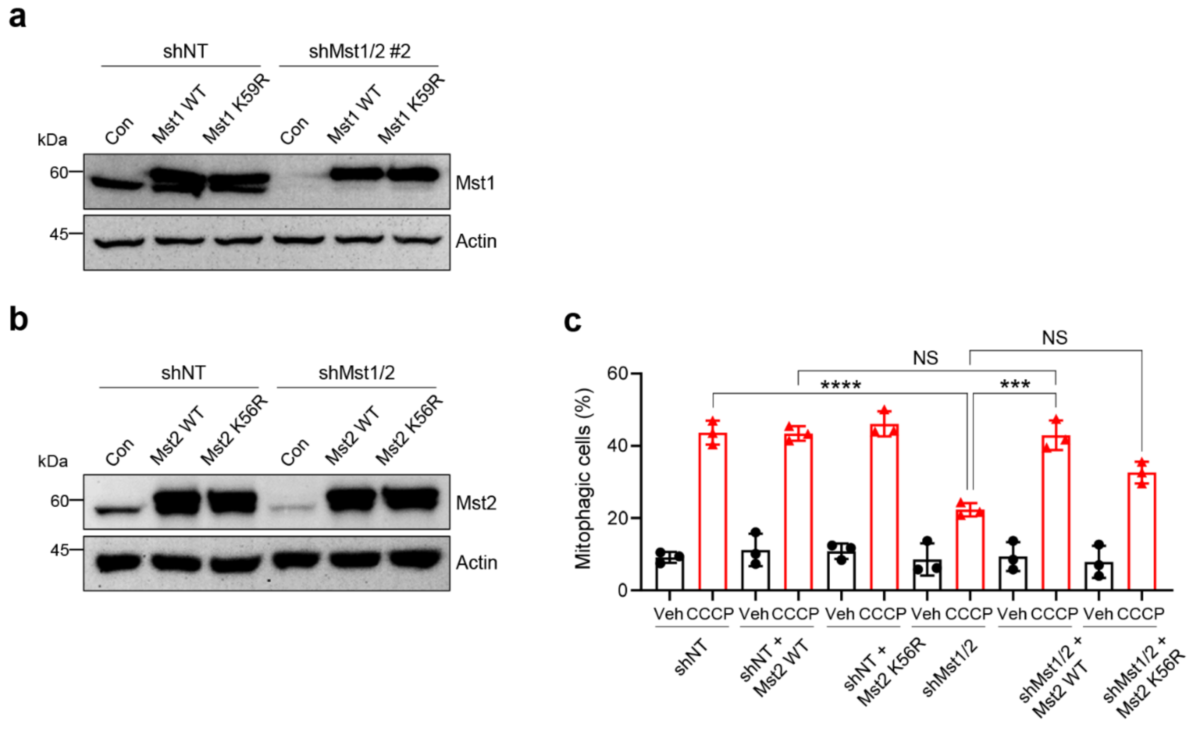

Supplementary Figure 2. Verification of the expression of wild-type Mst2 and the kinase dead form of Mst2 and their impact on mitophagy induction.

(a) HEK293 cells expressing mt-Keima and either control nontargeting shRNA (shNT) or Mst1 and Mst2 shRNAs (shMst1/2 #2) were transfected with Mst1 WT or Mst1 K59R expression plasmids. The cells were harvested 48 h after transfection and subjected to western blot analysis using the indicated antibodies. (b-c) HEK293 cells expressing mt-Keima and either control nontargeting shRNA (shNT) or Mst1 and Mst2 shRNAs (shMst1/2) were transfected with Mst2 WT or Mst2 K56R expression plasmids. Cells were harvested 48 h after transfection and subjected to western blot analysis using the indicated antibodies (b). After treatment with CCCP (10 μM) for 6 h, mitophagy levels were analyzed by flow cytometry (c). The results from three biological replicates are shown as the mean ± SD. Significance was determined by two-way ANOVA with Šidák's multiple-comparison test. ***P <0.001; ****P <0.0001. NS, not significant.

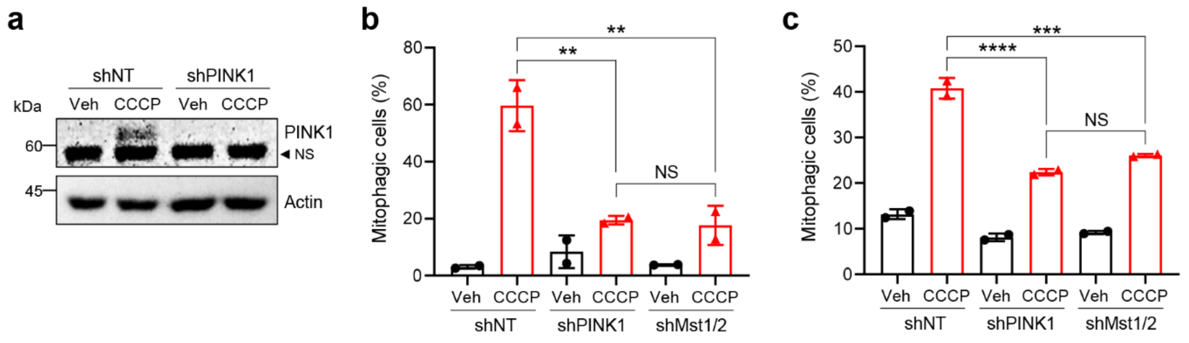

Supplementary Figure 3. Effect of PINK1 and shMst1/2 knockdown on mitophagy induction upon CCCP treatment.

(a) HEK293 cells expressing mt-Keima and either control nontargeting shRNA (shNT) or PINK1 shRNA (shPINK1) were treated with CCCP (10 μM) for 6 h, and cell lysates were subjected to western blot analysis using the indicated antibodies. NS, nonspecific band. (b-c) HeLa cells expressing Parkin (b) or SH-SY5Y cells (c) expressing either control nontargeting shRNA (shNT), PINK1 shRNA (shPINK1) or Mst1 and Mst2 shRNAs (shMst1/2) were treated with CCCP (10 μM) for 6 h, after which mitophagy was analyzed via flow cytometry. The results from two (a) or three biological replicates (b) are shown as the mean ± SD. Significance was determined by two-way ANOVA with Šidák's multiple-comparison test. **P <0.01; ***P <0.001; ****P <0.0001. NS, not significant.

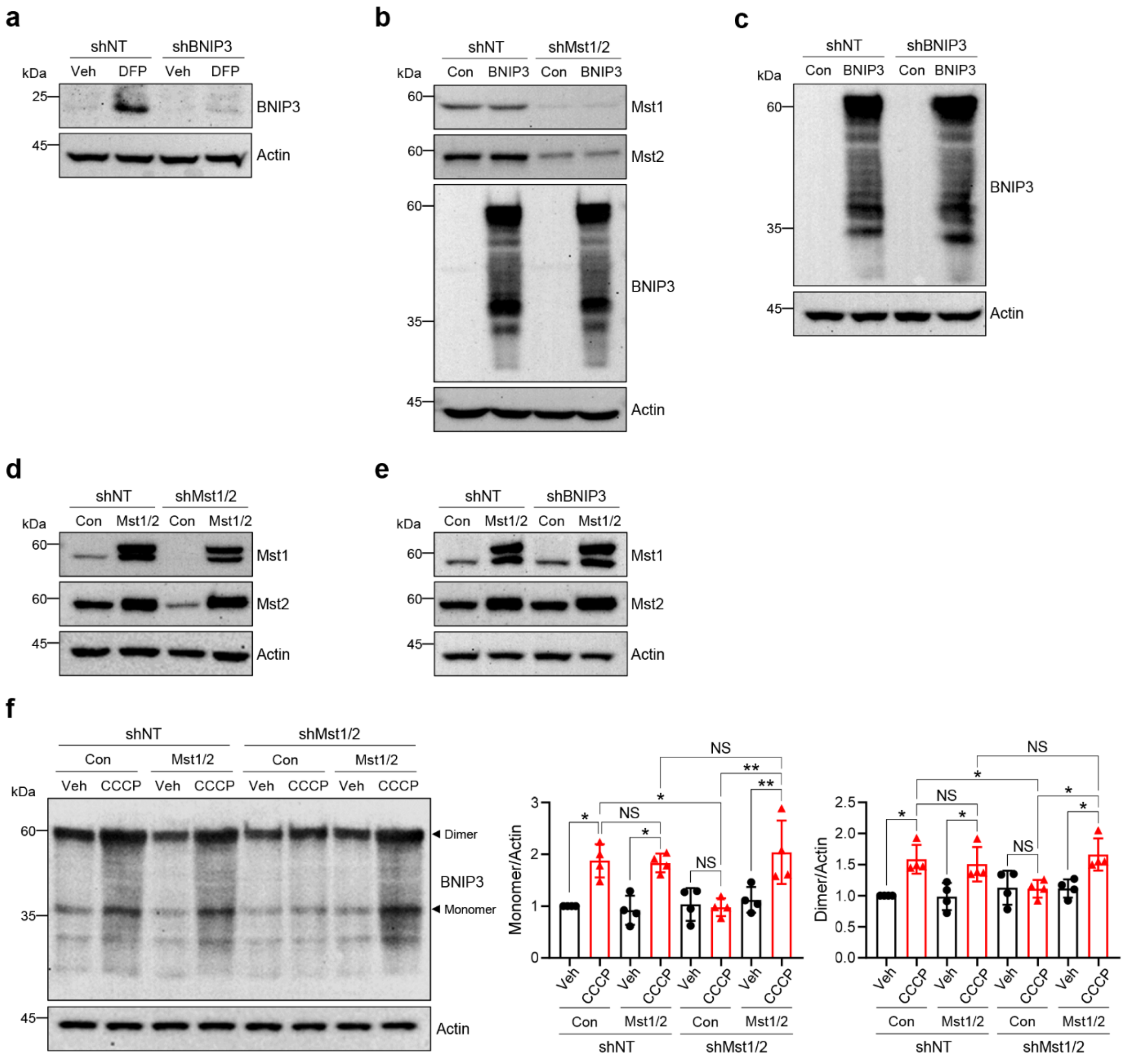

Supplementary Figure 4. Verification of BNIP3 or Mst1/2 knockdown and expression. (a) HEK293 cells expressing mt-Keima and either control nontargeting shRNA (shNT) or BNIP3 shRNA (shBNIP3) were treated with DFP (1 mM) for 24 h, and cell lysates were subjected to western blot analysis using the indicated antibodies. (b-e) HEK293 cells expressing mt-Keima and either control nontargeting shRNA (shNT), Mst1 and Mst2 shRNAs (shMst1/2) or BNIP3 shRNA (shBNIP3) were transfected with BNIP3 (b, c) or Mst1 and Mst2 (d, e) expression plasmids. Cells were harvested 48 h after transfection and subjected to western blot analysis using the indicated antibodies. (f) HEK293 cells expressing mt-Keima and either control nontargeting shRNA (shNT), Mst1 and Mst2 shRNAs (shMst1/2) were transfected with Mst1, Mst2 and BNIP3 expression plasmids, and cell lysates were subjected to western blot analysis using the indicated antibodies. The results from four biological replicates are shown as the mean ± SD. Significance was determined by two-way ANOVA with Šidák's multiple-

comparison test. $^{*}P < 0.05$; $^{**}P < 0.01$. NS, not significant.

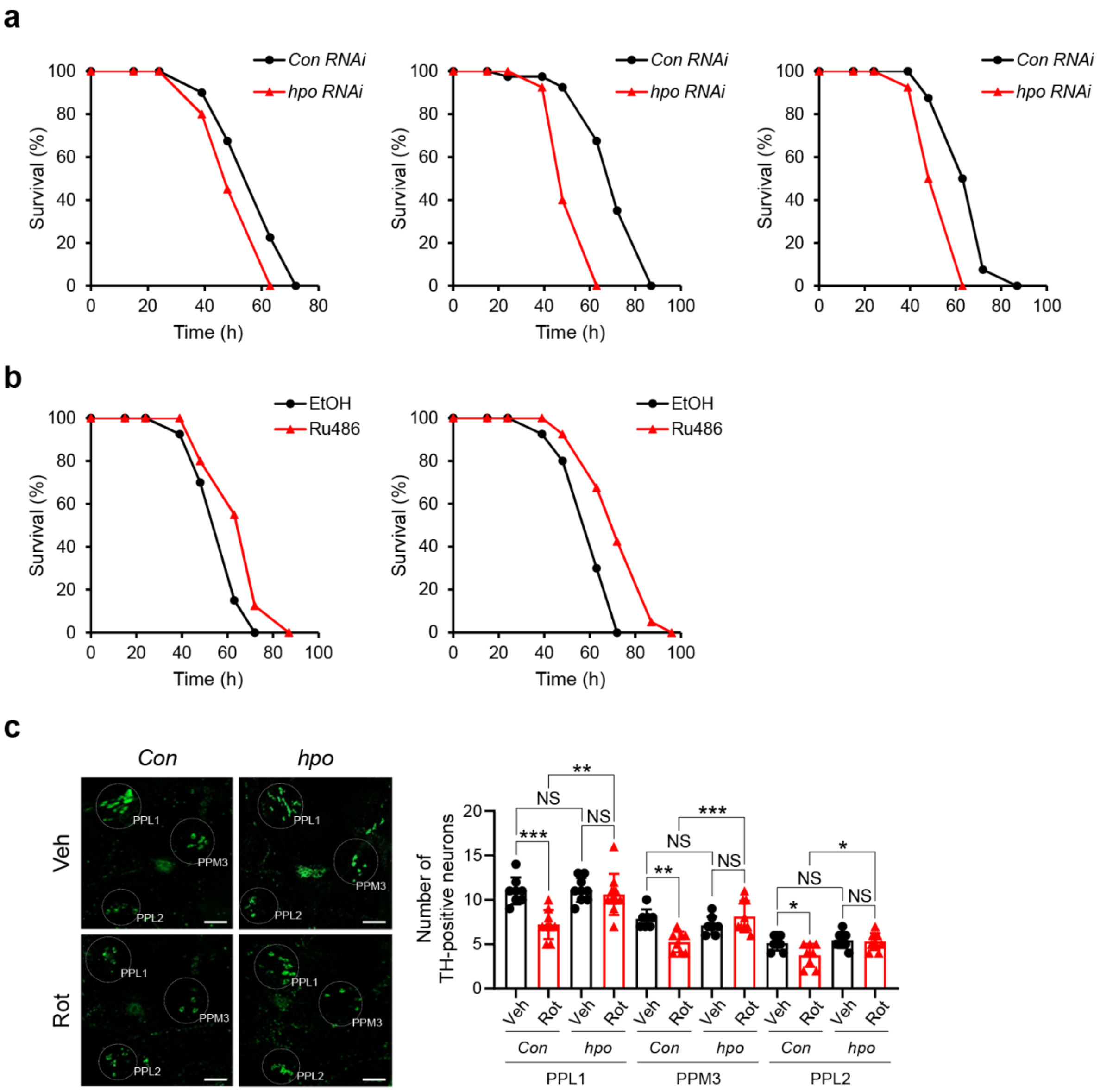

Supplementary Figure 5. Analysis of *Drosophila* survival upon rotenone treatment

(a) Repeated *Drosophila* survival assays upon rotenone treatment shown in Figure 5c. (b) Repeated *Drosophila* survival assays upon rotenone treatment shown in Figure 5f. (c) Seven-day-old *Drosophila* expressing mitoGFP (*TH>mitoGFP*) alone or in combination with *hippo* (*hpo*) (*TH>mitoGFP, hpo*) were treated with DMSO (Veh) or rotenone (Rot, 7.5 mM) for 48 h, and the number of dopaminergic neurons in the brain was analyzed via confocal microscopy. The quantified results are shown on the right as the mean ± SD (n ≧ 7 per sample). Scale bars: 50 µm. Significance was determined by two-way ANOVA with Šidák's multiple-comparison test. *P <0.05; **P <0.01; ***P <0.001. NS, not significant.

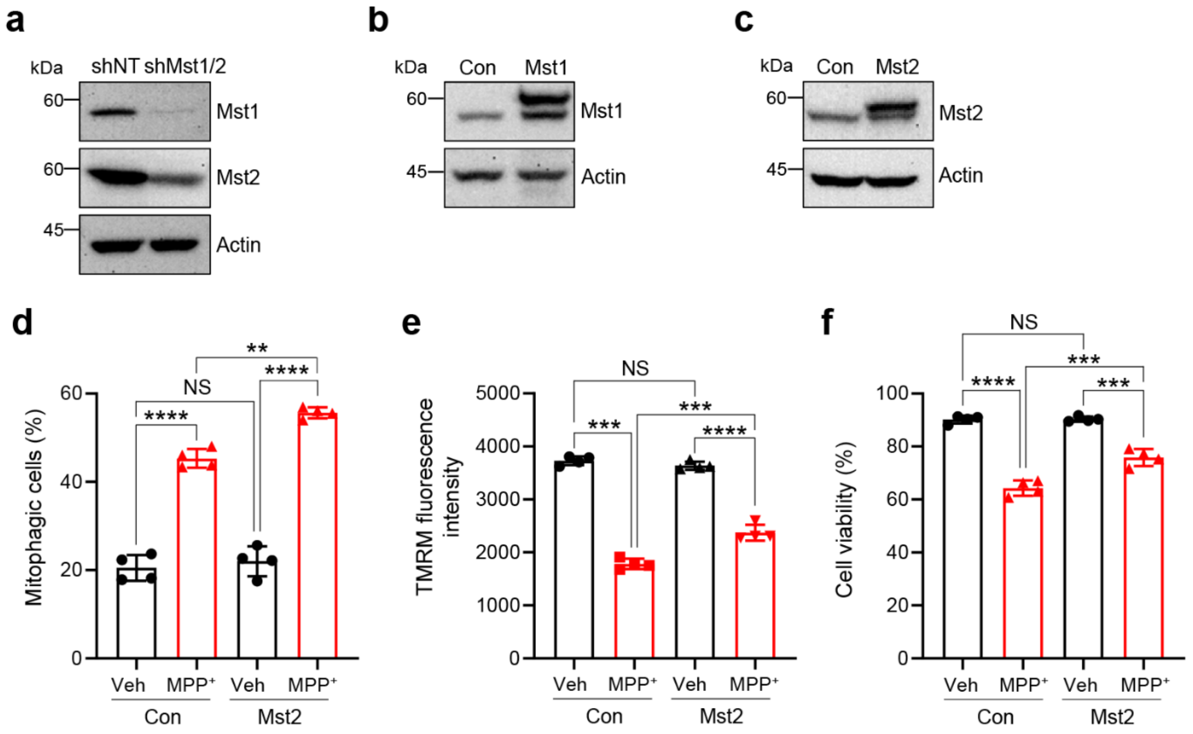

Supplementary Figure 6. Analysis of Mst1 expression in SH-SY5Y cells

(a) SH-SY5Y cells expressing mt-Keima and either control nontargeting shRNA (shNT) or Mst1 and Mst2 shRNAs (shMst1/2) were subjected to western blot analysis using the indicated antibodies. (b, c) SH-SY5Y cells were transfected with either vector (Con), Mst1 (b) or Mst2 (c) expression plasmid, and western blotting analysis was performed using the indicated antibodies. (d-f) SH-SY5Y cells were transfected with either vector (Con) or Mst2 expression plasmid and treated with MPP^+ (500 μM) for 24 h. Mitophagy levels were analyzed by flow cytometry (d). Mitochondrial membrane potential was analyzed by TMRM staining (e), and cell viability was analyzed (f). The results from four biological replicates are shown as the mean ± SD. Significance was determined by two-way ANOVA with Šidák’s multiple-comparison test. ***P* <0.01; ****P* <0.001; *****P* <0.0001. NS, not significant.

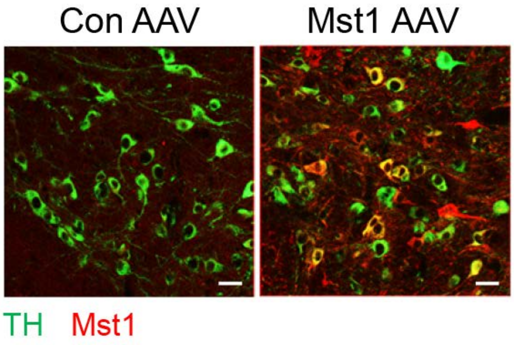

Supplementary Figure 7. Verification of Mst1 expression in mouse substantia nigra upon Mst1 AAV injection

C57 mice were injected with either control AAV (Con AAV) or Mst1 AAV into the substantia nigra of the right hemisphere. After four weeks of AAV injection, mouse midbrain sections were stained with anti-TH antibody (green) and anti-Mst1 antibody (red). Scale bars: 20 μm.
